# Long non-coding RNA PCED1B antisense RNA 1 promotes gastric cancer progression via modulating microRNA-215-3p / C-X-C motif chemokine receptor 1 axis

**DOI:** 10.1080/21655979.2021.1971503

**Published:** 2021-09-13

**Authors:** Junyu Ren, Ning Xu, Ruize Zhou, Fengchang Huang, Hongbin Zhang, Wenliang Li

**Affiliations:** Department of Oncology, The First Affiliated Hospital of Kunming Medical University, Kunming, China

**Keywords:** GC, CXCR1, PCED1B-AS1, miR-215-3p

## Abstract

Long non-coding RNAs (lncRNAs) emerge as vital modulators and tissue-specific biomarkers of multiple cancers, including gastric cancer (GC). Instead, the expression characteristics, biological function and molecular mechanism of lncRNA PCED1B antisense RNA 1 (PCED1B-AS1) in GC await more elaboration. In this study, 48 cases of GC tissues and matched non-cancerous tissues were collected, and PCED1B-AS1, microRNA-215-3p (miR-215-3p) and C-X-C motif chemokine receptor 1 (CXCR1) expression levels were detected by qRT-PCR. Besides, CCK-8, EdU, Transwell and Western blot assays were conducted to assess the impact of PCED1B-AS1 or miR-215-3p on cell growth, migration, invasion and epithelial-mesenchymal transition (EMT). The interaction between genes was verified by bioinformatics analysis, rna immunoprecitipation (RIP) and dual-luciferase reporter gene assays. We demonstrated that, PCED1B-AS1 expression level was raised in GC tissues and cell lines, and increased expression of PCED1B-AS1 was in association with tumor size, TNM stage and lymph node metastasis in GC patients. Additionally, PCED1B-AS1 overexpression promoted GC cells proliferation, migration, invasion and EMT, and miR-215-3p overexpression counteracted the biological effects of PCED1B-AS1. Mechanistically, PCED1B-AS1 specifically inhibited miR-215-3p expressions, thus up-regulating CXCR1 expressions. In conclusion, PCED1B-AS1 accelerates GC progression via adsorbing miR-215-3p and up-regulating CXCR1, indicating that PCED1B-AS1 is a novel therapeutic target for treating GC.

## Introduction

Gastric cancer (GC) is a common malignancy, posing a threat to global public health [1]. GC is also one of the main causes of cancer-related deaths in China [2]. Although there is great progression in diagnosis and treatment in recent years, patients who suffered from advanced or metastatic GC still have poor prognosis [3,4]. Thus, searching for novel biomarkers and understanding the potential mechanism of GC are urgent for the improving the diagnosis and prognosis of GC.

Non-coding RNAs (ncRNAs) are widely expressed in human cells, including microRNAs (miRNAs) with approximately 22*nt* and long non-coding RNAs (lncRNAs) with exceeding 200*nt*, and they all feature prominently in post-transcriptional regulation [5]. LncRNA is at play in various biological activities of tumor cells, including proliferation, apoptosis, metastasis, and drug resistance [6–8]. To date, diverse abnormally expressed lncRNAs have been unveiled in GC [9]. LncRNA, as a competitive endogenous RNA (ceRNA), can modulate downstream target gene expressions via decoying miRNAs. For example, lncRNA LINC00511 represses miR-625-5p and up-regulates transducers and activators of transcription 3 (STAT3) to facilitate the progression of GC [10]. Another study reports that, lncRNA AL139002.1 promotes GC progression by sponging miR-490-3p to regulate hepatitis A virus cellular receptor 1 [11]. LncRNA PCED1B antisense RNA 1 (PCED1B-AS1) mediates macrophage apoptosis and autophagy via targeting the miR-155 axis in active tuberculosis [12]. Additionally, PCED1B-AS1 is a tumor-promoter, and for example, PCED1B-AS1 expression level is markedly raised in glioblastoma tissues and cell lines, which is in association with larger tumor size and higher grade [13]. Instead, the role of PCED1B-AS1 in GC is still unclear.

Chemokines, as a family of small proteins (8–11kDa), which are divided into four classes (C, CC, CXC, and CX3C), can promote directional chemotaxis of leukocytes and play important roles in inflammation and cancers [14]. CXC chemokines bind to the G-protein-coupled receptors (GPCR) e.g. C-X-C motif chemokine receptor 1 (CXCR1) and C-X-C motif chemokine receptor 2 (CXCR2), to exert their biological effects [15,16]. CXCR1 is reported to be up-regulated in GC tissues, and its high expression indicates poor prognosis of GC patients; besides, CXCR1 expedites the growth, migration, and invasion of GC cells [17,18]. Instead, its regulatory mechanism in GC is not yet clear.

In this work, we supposed that PCED1B-AS1 could probably participate in GC progression. Our study was performed to investigate the expression characteristics, biological function and underlying mechanism of PCED1B-AS1 in GC. We demonstrated that, PCED1B-AS1 expression level was markedly up-regulated in GC tissues, which was closely associate with larger tumor size, higher TNM stage and lymph node metastasis. Also, PCED1B-AS1 strengthened cell viability, migration and invasion, and epithelial-mesenchymal transition (EMT) through the microRNA-215-3p (miR-215-3p)/CXCR1 axis.

## Materials and methods

### Clinical samples collection

48 pairs of GC tissues and normal tissues adjacent to cancer were available from the First Affiliated Hospital of Kunming Medical University. Besides, this work was endorsed by the Ethics Committee of the First Affiliated Hospital of Kunming Medical University with informed consent available from patients. All volunteers did not receive any treatment before operation, and the clinicopathological characteristics of the sufferers are detailed in Table 1. Immediately after the removal, the tissue samples were positioned in liquid nitrogen and subsequently stored at −80°C.

**Table 1.** The correlations of PCED1B-AS1 with clinicopathological features of patients with gastric cancer

Characteristics	Patients(n)	PCED1B-AS1expression		*P* values
		Low expression (n = 21)	High expression (n = 27)	
Age (years)				
≤ 60	32	12	20	0.217
> 60	16	9	7	
Tumor size (cm)				
≤ 5	16	11	5	0.014*
> 5	32	10	22	
Gender				
Male	31	12	19	0.342
Female	17	9	8	
TNM stage				
I – II	27	16	11	0.014*
III–IV	21	5	16	
Tumor differentiation				
Well + moderate	29	14	15	0.435
Poor	19	7	12	
Lymph node metastasis				
Absent	28	17	11	0.005**
Present	20	4	16	
Distant metastasis				
Absent	21	8	13	0.486
Present	27	13	14	

*P < 0.05.

### Cell culture and transfection

The Cell Bank of Chinese Academy of Sciences (Shanghai, China) was the supplier of four human GC cell lines (HGC-27, KATO III, NCI-N87, and AGS), normal gastric epithelial cell line (GES-1) and the human embryo kidney epithelial cell line HEK293T. The cells were cultured in Roswell Park Memorial Institute-1640 medium (Invitrogen, Carlsbad, CA, USA) with 10% fetal bovine serum (FBS; Thermo Fisher Scientific, Waltham, MA, USA), 100 units/ml penicillin and 100 μg/ml streptomycin (Invitrogen, Carlsbad, CA, USA) in 5% CO_2_ at 37°C.

Small interfering RNA (siRNA) targeting PCED1B-AS1 (si-PCED1B-AS1-1, si-PCED1B-AS1-2 and si-PCED1B-AS1-3) and negative control (si-NC), miR-215-3p-mimics/NC-mimics, miR-215-3p inhibitor/NC inhibitor, PCED1B-AS1 overexpression vector (pc-PCED1B-AS1), CXCR1 overexpression vector (pc-CXCR1)/pcDNA empty vector (Vector) were available from GenePharma (Shanghai, China). The GC cells were subsequently transfected with the plasmids or the oligonucleotides by Lipofectamine 3000 (Invitrogen, Carlsbad, CA, USA) as protocols, and 48 h later, quantitative real-time polymerase chain reaction (qRT-PCR) was executed to estimate the transfection efficiency.

### qRT-PCR

The total RNA from GC tissues and cell lines was extracted by a RNAiso Plus kit (TaKaRa, Dalian, China) and reverse-transcribed into complementary DNA (cDNA) with a PrimeScript First Strand cDNA Synthesis Kit (Takara, Dalian, China). Next, qRT-PCR amplifications were accomplished by a SYBR® Premix ExTaq kit (TaKaRa, Dalian, China) on ABI PRISM 7300 system (Applied Biosystems, Foster City, CA, USA). Besdes, Glyceraldehyde-3-phosphate dehydrogenase (GAPDH) was adopted as the internal reference for PCED1B-AS1 and CXCR1, and U6 snRNA as that for miR-215-3p. The relative expressions of the genes involved in this study were calculated by 2^−ΔΔCt^. Specifically, the primer sequences are listed in Table 2. To identify the sub-cellular localization of PCED1B-AS1 in GC cells, nuclear/cytoplasmic separation of GC cells was performed with a PARIS™ Kit (Ambion, Austin, TX, USA) according to the manufacturer’s instruction. After the cytoplasmic RNA and nuclear RNA were isolated, PCED1B-AS1 level was measured by qRT-PCR, with GAPDH as the cytoplasmic endogenous control and the U6 as the nuclear endogenous control.

**Table 2.** The primer sequence used in this study

gene	primer sequence
PCED1B-AS1	Forward: 5ʹ-TCAAGCCAATCAGCTGACAC-3’
	Reverse: 5ʹ- AAACAAATGCCCTGCTTGAC-3’
CXCR1	Forward: 5ʹ-CTGACCCAGAAGCGTCACTTG −3’
	Reverse: 5ʹ-CCAGGACCTCATAGCAAACTG-3’
miR-215-3p	Forward: 5ʹ-TGGATTTGGACGCATTGGTC-3’
	Reverse: 5ʹ-TTTGCACTGGTACGTGTTGATA-3’
GAPDH	Forward: 5ʹ-ACCCAGAAGACTGTGGATGG-3’
	Reverse: 5ʹ- TTCAGCTCAGGGATGACCTT-3’
U6	Forward: 5ʹ-TGCGGGTGCTCGCTTCGGCAGC-3’
	Reverse: 5ʹ-CCAGTGCAGGGTCCGAGGT-3’

### Cell counting kit-8 (CCK-8) assay

GC cells were positioned in 96-well plates at 2 × 10^3^ cells per well, and cultured. At the 24^th^, 48^th^, and 72^nd^ h, 10 μL of CCK-8 reagent (Dojindo Molecular Technologies, Japan) was added into each well respectively, and the cells were cultured at 37°C for 2 h. Then the OD_450 nm_ value was estimated by a microplate reader (Bio-Rad, Hercules, CA, USA).

### EdU proliferation assay

Cell proliferation was examined with an EdU assay kit (RiboBio, Guangzhou, China). Cells were cultured with 50 μM EdU for 2 h, then were fixed in 4% paraformaldehyde and subsequently stained with Apollo staining solution away from light for 2 h at room temperature. Subsequently, the cells were stained with DAPI staining solution (Beyotime, Shanghai, China) away from light for 30 min at room temperature. Next, the cells were washed with phosphate buffer saline (PBS), the EdU positive cells were photographed under a fluorescence microscopy (Olympus, Tokyo, Japan) in five randomly selected fields, and counted.

### Cell migration and invasion assay

48 h after transfection, cells in serum-free medium were transferred into the upper compartment of Transwell chambers (pore size: 8 μm, Millipore, Bedford, MA, USA). 600 μL of medium containing 10% FBS was dripped into the lower compartment. After the cells were cultured for 24 h, the cells which passed through membrane were fixed with 4% formaldehyde and stained with 0.5% crystal violet. Ultimately, cells were observed and the numbers were subsequently counted under the microscope (Olympus, Tokyo, Japan). Notably, Matrigel (Sigma-Aldrich, Louis, MO, USA) was used to cover the membrane to mimic the extracellular matrix in invasion analysis, but it was not used in migration assay.

### Dual-luciferase reporter assay

The sequences of PCED1B-AS1 or CXCR1 3ʹUTR containing the wild type (WT) or mutant type (MUT) miR-215-3p binding sites were cloned into the pmirGLO luciferase reporter vector (Promega, Madison, WI, USA) to generate the reporter plasmids (PCED1B-AS1-WT, CXCR1-WT, PCED1B-AS1-MUT and CXCR1-MUT). HEK293T cells were inoculated in 96-well plate and cultured for 24 h, then co-transfected with the reporter plasmids and miR-215-3p mimics or miR-NC by Lipofectamine 3000 (Invitrogen, Carlsbad, CA, USA). 48 h later, the luciferase activity was measured by the Dual-Luciferase Reporter System (Promega, Madison, WI, USA).

### Western blot assay

Total proteins in tissues and cells were respectively extracted by RIPA lysis buffer (Beyotime, Shanghai, China), and protein concentration was detected by a bicinchoninic acid (BCA) kit (Beyotime, Shanghai, China). The same amount of protein in each group was subsequently separated by SDS-PAGE and then transferred to polyvinylidene fluoride membranes (Millipore, Billerica, MA, USA). Subsequently, the membranes was blocked by 5% skimmed milk for 2 h at room temperature, and then incubated at 4°C overnight with primary antibodies anti-E-cadherin (1:1000; ab40772; Abcam), anti-N-cadherin (1:1000; ab18203; Abcam), anti-Vimentin (1:1000; ab92547; Abcam), CXCR1 (1:1000; ab124344; Abcam) and anti-GAPDH (1:1000; ab9485; Abcam), and incubated with Goat Anti-Rabbit IgG H&L (HRP) (1:2000; ab205718; Abcam) secondary antibody for 1 h. Besides, the protein bands were developed by an electrochemiluminescence kit (Pierce Biotechnology, Rockford, IL, USA), with GAPDH as the internal control.

### RNA immunoprecipitation (RIP) assay

A Magna RIP RNA-Binding Protein Immunoprecipitation Kit (Millipore, Billerica, MA, USA) was utilized for RIP assay. Cell was lysed with RIP lysis buffer, and cell lysate was incubated with human anti-Argonaute 2 (Ago 2) antibody (Millipore, Billerica, MA, USA) or negative control mouse IgG (Millipore, Billerica, MA, USA) coupled with magnetic bead overnight at 4°C. Then the proteins in the immunoprecipitate were degraded with proteinase K and the immunoprecipitated RNA was then isolated and purified, followed by qRT-PCR detection.

### Statistical analysis

SPSS 22.0 software (SPSS, Inc., Chicago, IL, USA) and GraphPad Prism 8 (GraphPad Software, Inc., La Jolla, CA, USA) were employed for statistical analysis. Each experiment was repeated for three times or more, with the data expressed as the mean ± standard deviation (SD). Additionally, student’s *t*-test and one-way analysis of variance (ANOVA) were performed for comparisons. Chi-squared test was executed to analyze the association between PCED1B-AS1 expression and the clinicopathological characteristics. Besides, Spearman’s correlation analysis was used to analyze the correlation between gene expressions in GC tissues. Statistically, *P* < 0.05 is significant.

## Results

In the present study, with *in vitro* experiments, we investigate the expression pattern and biological functions of PCED1B in GC. We also investigated the regulatory effects of PCED1B-AS1 on the expression of miR-215-3p and CXCR1.

### 1. PCED1B-AS1 is highly expressed in GC tissues and cell lines

**Figure 1.**
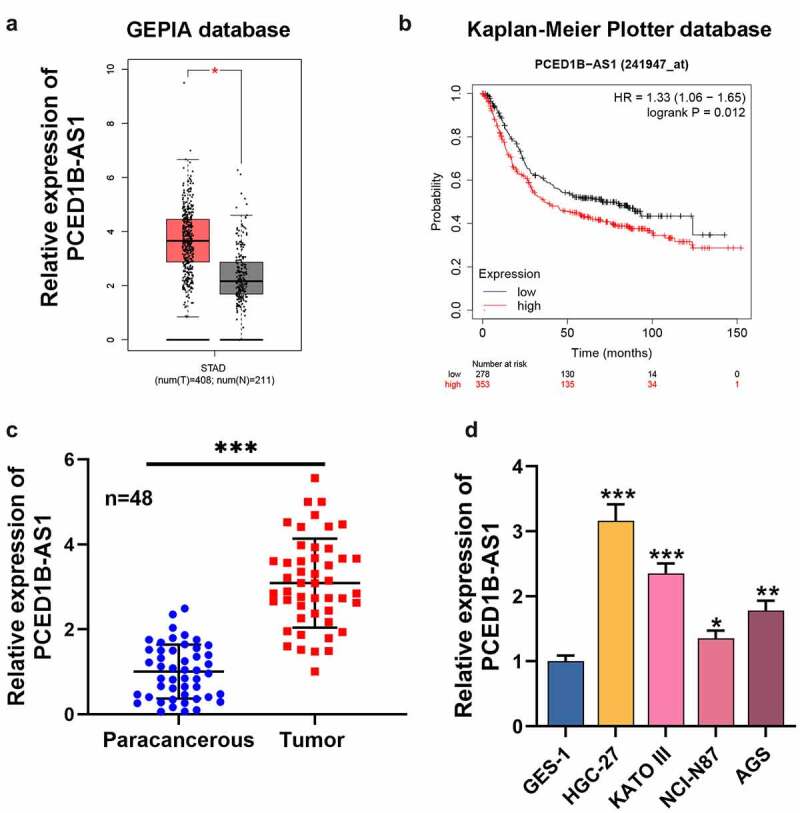
PCED1B-AS1 is highly expressed in GC tissues and cell lines

First of all, we searched GEPIA database (http://gepia.cancer-pku.cn/) and observed that PCED1B-AS1 expression in GC patients was demonstrably higher than that in normal tissues (Figure 1a). Besides, Kaplan-Meier Plotter database (http://kmplot.com/analysis/index.php?p=service) highlighted that the overall survival of patients with low PCED1B-AS1 expression was longer than that of patients with high PCED1B-AS1 expression (Figure 1b). qRT-PCR revealed that PCED1B-AS1 expression in GC tissues was significantly higher compared with that in normal tissues adjacent to cancer (Figure 1c). In addition, as against normal gastric epithelium cell line GES-1, PCED1B-AS1 expression in GC cell lines (HGC-27, KATO III, NCI-N87, and AGS) was dramatically increased (Figure 1d). Additionally, it was also revealed that higher PCED1B-AS1 expression in GC tissues was associated with larger tumor size, higher TNM stage and lymph node metastasis (Table 1).

A. The expression of PCED1B-AS1 in the tissues of GC patients was analyzed by GEPIA database.B. The relationship between the expression of PCED1B-AS1 and the the overall survival time of GC patients was analyzed by Kaplan-Meier Plotter database.C. The expression of PCED1B-AS1 was detected by qRT-PCR in GC and normal tissues.D. The expression of PCED1B-AS1 in GES-1 cells and GC cell lines (HGC-27, KATO III, NCI-N87 and AGS) was detected by qRT-PCR.*P < 0.05, ** *P* < 0.01, and *** *P* < 0.001

### 2. Effects of PCED1B-AS1 on GC cell viability, migration, invasion and EMT

**Figure 2.**
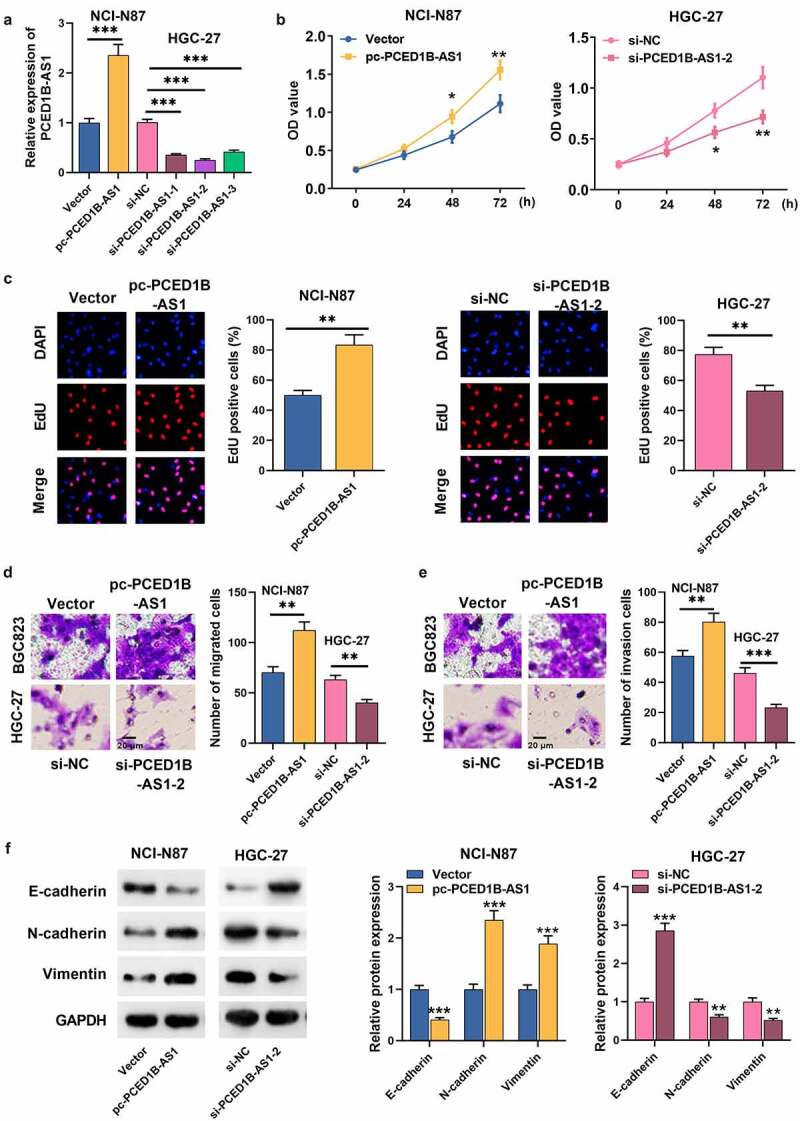
The effect of PCED1B-AS1 on GC cells’ proliferation, migration, invasion and EMT

As mentioned above, among the GC cells, PCED1B-AS1 expression was the highest in HGC-27 and the lowest in NCI-N87, so we transfected siRNA (si-PCED1B-AS1-1, si-PCED1B-AS1-2, and si-PCED1B-AS1-3) and PCED1B-AS1 overexpression vector (pc-PCED1B-AS1) targeting PCED1B-AS1 into HGC-27 and NCI-N87, respectively (Figure 2a). CCK-8 and EdU assays revealed that the proliferation rate of GC cells with PCED1B-AS1 overexpression was higher, while PCED1B-AS1 knockdown worked oppositely (Figure 2b-c). Transwell assay revealed that the metastatic potential of GC cells with PCED1B-AS1 overexpression was evidently stronger than that of the control group, and PCED1B-AS1 knockdown suppressed the migration and invasion of GC cells (Figure 2d-e). Additionally, Western blot assay depicted that PCED1B-AS1 overexpression promoted N-cadherin and Vimentin expressions and inhibited the protein expression of E-cadherin, while PCED1B-AS1 knockdown had the opposite effect (figure 2f).

A. The expression of PCED1B-AS1 in GC cells transfected with si-PCED1B-AS1 and pc-PCED1B-AS1 was detected by qRT-PCR.B-C. CCK-8 and EdU methods were used to detect the regulatory effect of PCED1B-AS1 on the proliferation of GC cells.D-E. Transwell assay was used to detect the regulatory effect of PCED1B-AS1 on migration and invasion of GC cells.F. Western blot assay was used to detect the regulatory effect of PCED1B-AS1 on protein levels of EMT markers (E-cadherin, N-cadherin and Vimentin) in GC cells.* *P* < 0.05, ** *P* < 0.01, and *** *P* < 0.001.

### 3. PCED1B-AS1 works as a ceRNA via adsorbing miR-215-3p in GC

**Figure 3.**
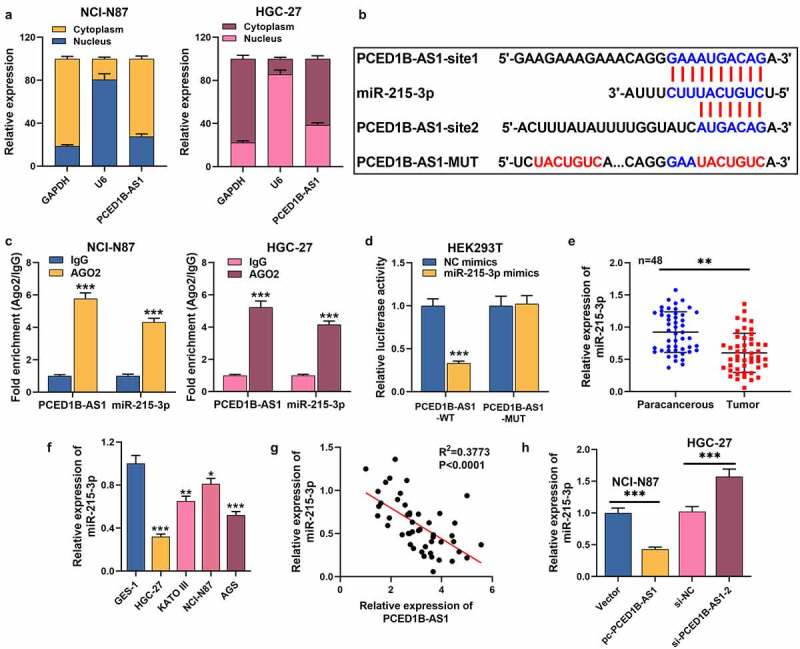
PCED1B-AS1 serves as a ceRNA by sponging miR-215-3p in GC

To expound the hidden regulatory mechanism of PCED1B-AS1 in GC, we first performed nucleocytoplasmic separation experiments and observed that PCED1B-AS1 was mainly distributed in the cytoplasm of GC cells (Figure 3a). DIANA database (http://carolina.imis.athena-innovation.gr/diana_tools/web/index.php?r=lncbasev2/index-predicted) highlighted that miR-215-3p may be a promising downstream target of PCED1B-AS1 (Figure 3b). In the 48 patients, low miR-215-3p expression was associated with larger tumor size, higher TNM stage and poor differentiation status of the tumor tissues (Table 3). RIP assay revealed that PCED1B-AS1 and miR-215-3p were specifically enriched in anti-Ago2 group, but not in anti-IgG group (Figure 3c). Dual-luciferase reporter gene assay depicted that the luciferase activity of HEK293T cells co-transfected with PCED1B-AS1-WT and miR-215-3p mimics was greatly lower than that of NC mimics group, but there was no significant change in PCED1B-AS1-MUT group (Figure 3d). Besides, miR-215-3p was markedly reduced in GC tissues and cell lines (Figure 3e-f). Furthermore, miR-215-3p expression was negatively correlated with PCED1B-AS1 expression in GC tissues (Figure 3g). Additionally, miR-215-3p was inhibited in GC cells with PCED1B-AS1 overexpression, but overexpressed in cell lines with PCED1B-AS1 knockdown (Figure 3h). These highlights that PCED1B-AS1, as a miRNA sponge, has a negative regulatory effect on miR-215-3p expression.

**Table 3.** The correlations of miR-215-3p with clinicopathological features of patients with gastric cancer

Characteristics	Patients(n)	miR-215-3p expression		*P* values
		Low expression (*n* = 17)	High expression (*n* = 31)	
Age (years)				
≤60	32	11	21	0.831
>60	16	6	10	
Tumor size (cm)				
≤5	16	4	12	0.037*
>5	32	13	9	
Gender				
Male	31	13	18	0.202
Female	17	4	13	
TNM stage				
I – II	27	6	21	0.030*
III–IV	21	11	10	
Tumor differentiation				
Well + moderate	29	4	25	<0.001***
Poor	19	13	6	
Lymph node metastasis				
Absent	28	7	21	0.074
Present	20	10	10	
Distant metastasis				
Absent	21	6	15	0.382
Present	27	11	16	

*P < 0.05, and *** *P* < 0.001.

A. The expression of PCED1B-AS1 in the nuclear fraction and cytoplasmic fraction of GC cells was detected by qRT-PCR. U6 and GAPDH were detected in nuclear and cytoplasmic fractions.B. Potential binding sites between PCED1B-AS1 and miR-215-3p were analyzed through the LncBase Predict v.2 database.C. RIP assay was used to validate the interaction between PCED1B-AS1 and miR-215-3p.D. The targeting relationship between PCED1B-AS1 and miR-215-3p was confirmed by dual-luciferase reporter gene assay.E. qRT-PCR was used to detect the expression of miR-215-3p in 48 cases of GC tissues and normal adjacent tissues.F. The expression of miR-215-3p in GES-1 cells and GC cell lines (HGC-27, KATO III, NCI-N87 and AGS) was detected by qRT-PCR.G. Spearman’s correlation analysis was used to analyze the correlation between PCED1B-AS1 expression and miR-215-3p expression in tissues.H. qRT-PCR was used to detect the expression of miR-215-3p in GC cells, after PCED1B-AS1 was overexpressed or knocked down.*P < 0.05, ** *P* < 0.01, and *** *P* < 0.001.

### 4. CXCR1 emerges as a target of miR-215-3p

**Figure 4.**
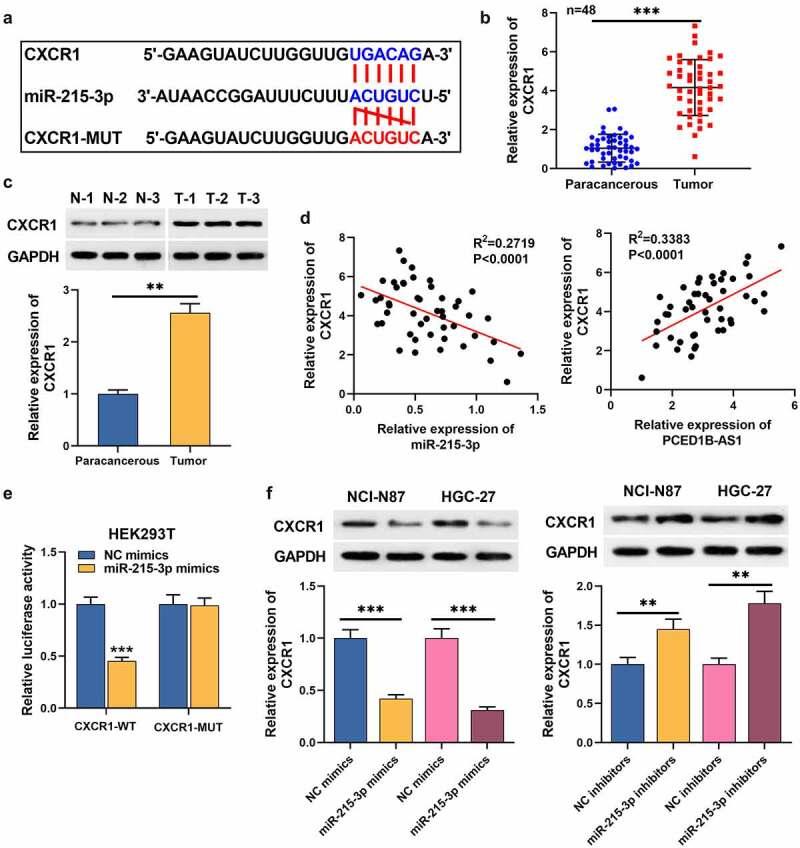
CXCR1 is a target mRNA of miR-215-3p

TargetScan database (http://www.targetscan.org/vert_71/) predicted that miR-215-3p had a putative binding site in CXCR1 3ʹUTR (Figure 4a). qRT-PCR and Western blot assays uncovered that CXCR1 expression in GC tissues was higher than that in adjacent normal tissues (Figure 4b-c). In the 48 patients, higher CXCR1 expression was associated with the distant metastasis after surgery (Table 4). Notably, CXCR1 expression was negatively interrelated with miR-215-3p expression in GC tissues, but positively interrelated with PCED1B-AS1 expression (Figure 4d). Dual-luciferase reporter gene assay showed that miR-215-3p mimics remarkably repressed the luciferase activity of CXCR1-WT (Figure 4e). Western blot assay uncovered that miR-215-3p could inhibit CXCR1 expression in GC cells (figure 4f). These data suggests that CXCR1 is the target of miR-215-3p.

**Table 4.** The correlations of CXCR1 with clinicopathological features of patients with gastric cancer

Characteristics	Patients(n)	CXCR1 expression		*P* values
		Low expression (*n* = 20)	High expression (*n* = 28)	
Age (years)				
≤60	32	14	18	0.679
>60	16	6	10	
Tumor size (cm)				
≤5	16	8	8	0.408
>5	32	12	20	
Gender				
Male	31	13	18	0.959
Female	17	7	10	
TNM stage				
I – II	27	11	16	0.883
III–IV	21	9	12	
Tumor differentiation				
Well + moderate	29	14	15	0.251
Poor	19	6	13	
Lymph node metastasis				
Absent	28	12	16	0.843
Present	20	8	12	
Distant metastasis				
Absent	21	13	8	0.012*
Present	27	7	20	

*P < 0.05 Statistically significant.

A. The potential binding site between miR-215-3p and CXCR1 was predicted by TargetScan database.B-C. The expression of CXCR1 was detected by qRT-PCR and Western blot assays. N, normal tissues; T, tumor tissues.D. The correlation between CXCR1 and the expression of miR-215-3p and PCED1B-AS1 was analyzed by Spearman’s correlation analysis.E. The targeting relationship between CXCR1 and miR-215-3p was confirmed by dual-luciferase reporter assay.F. Western blot was used to detect the expression of CXCR1 protein in HGC-27 and NCI-N87 cells transfected with miR-215-3p mimics or inhibitors. **P < 0.01, and ***P < 0.001

### 5. PCED1B-AS1 facilitates the growth, migration, invasion, and EMT process of GC cells via regulating the miR-491-5p/CXCR1 axis

**Figure 5.**
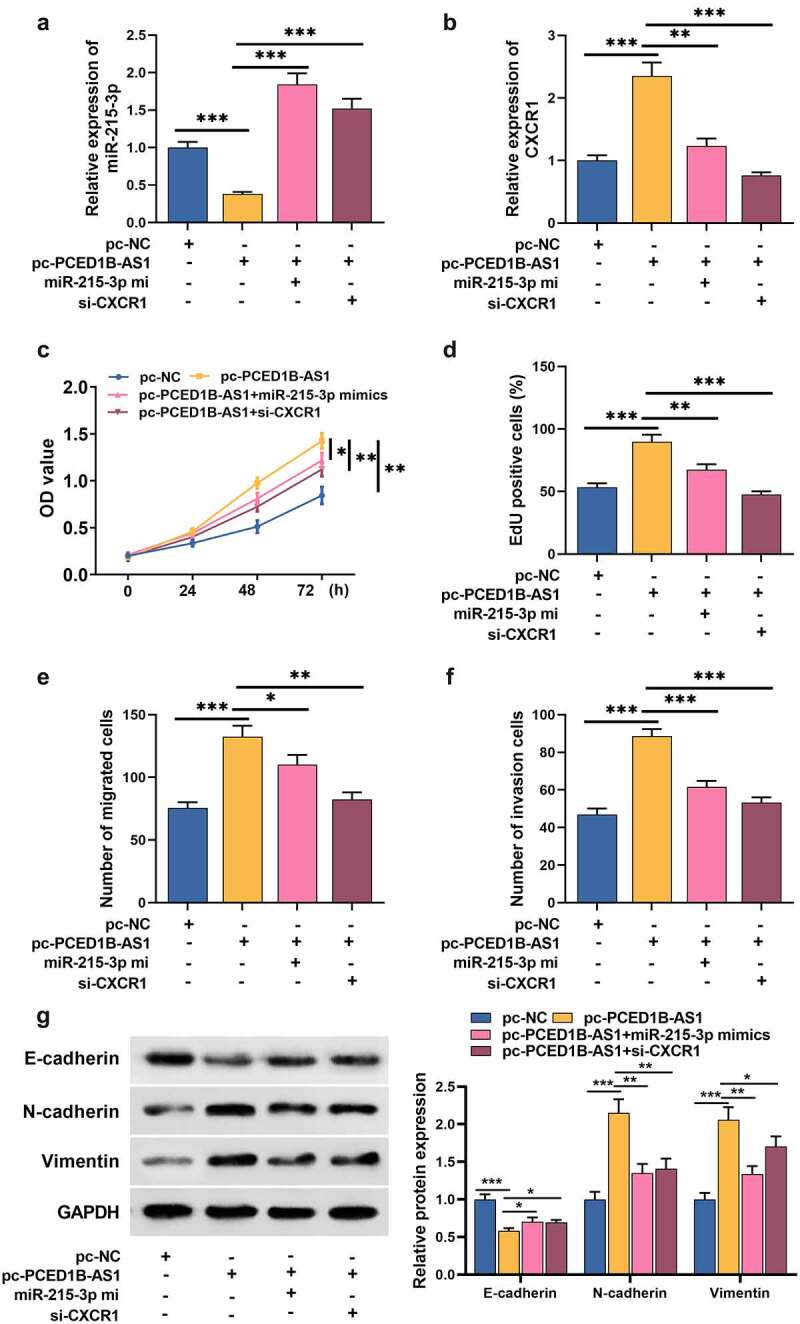
PCED1B-AS1 promotes the proliferation, migration, invasion and EMT of GC cells by regulating the miR-491-5p/CXCR1 axis

To confirm whether PCED1B-AS1 participates in the GC progression by adsorbing miR-215-3p and up-regulating CXCR1 expression, rescue assays were performed. MiR-215-3p mimic reversed the inhibitory effect of pc-PCED1B-AS1 on miR-215-3p, and PCED1B-AS1 overexpression could promote the expression of CXCR1, but the si-CXCR1 markedly inhibited CXCR1 expression (Figure 5a-b). Compared with GC cells transfected with pc-PCED1B-AS1, the growth of GC cells transfected with pc-PCED1B-AS1+ miR-215-3p mimics or pc-PCED1B-AS1+ si-CXCR1 was greatly inhibited (Figure 5c-d). Transwell and Western bolt assays revealed that pc-PCED1B-AS1 enhanced cell migration, invasion, and EMT process, while miR-215-3p mimics or si-CXCR1 transfection counterracted the effect of pc-PCED1B-AS1 on GC cells (Figure 5e-g).

A-B. MiR-215-3p mimic or si-CXCR1 was co-transfected with pc-PCED1B-AS1 into NCI-N87 cells, and the expression of miR-215-3p and CXCR1 detected by qRT-PCR.C-D. CCK-8 and EdU assays were used to detect the proliferation of GC cells after transfection.E-F. Transwell assay was used to detect the migration and invasion of GC cells after transfection.G. Western blot assay was used to detect the protein levels of E-cadherin, N-cadherin, and Vimentin in GC cells after transfection.*P < 0.05, ** *P* < 0.01, and *** *P* < 0.001.

## Discussion

Reportedly, lncRNA features prominently in regulating epigenetics, transcriptional and post-transcriptional processes to modulate gene expression. LncRNA, abnormally expressed in cancer cells, participates in regulating the progression of multiple cancers, including GC. For example, lncRNA cancer susceptibility 11 can promote GC development by facilitating cell cycle progression [19]. LncRNA SOX2 overlapping transcript is in high expression in GC, and it contributes to the growth and metastasis of GC cells [20]. There are few studies on the role of PCED1B-AS1 in tumors, specifically, PCED1B-AS1, as a tumor-promoter, partakes in modulating glioma cell proliferation and apoptosis [13]. Additionally, it also facilitates the progression of clear cell renal cell carcinoma and pancreatic cancer [21,22]. Here, we proved that PCED1B-AS1 expression was greatly raised in GC tissues, which was closely associated with larger tumor size, higher TNM stage and lymph node metastasis. Besides, functional experiments further confirmed that PCED1B-AS1 overexpression strengthened malignant biological behaviors of GC cells, while PCED1B-AS1 inhibition showed the opposite effects, implying that PCED1B-AS1 was a tumor-promoter in GC.

As reported, lncRNAs, as a ceRNA, can exert its function by sponging endogenous miRNA to inhibit mRNA translation, and this mechanism is involved in tumorigenesis [23,24]. For example, small nucleolar RNA host gene 5/microRNA-32/Kruppel like factor 4 (KLF4) axis regulates GC cell migration, which contributes to improving the diagnosis and treatment of GC [25]. LncRNA TMPO antisense RNA 1 can sponge miR-140-5p and indirectly regulate SRY-box transcription factor 4 expression to accelerate the progression of GC [26]. Reportedly, PCED1B-AS1 is elevated in gliomas and head and neck squamous cell carcinoma [13,27], and PCED1B-AS1 modulates the multiplication and apoptosis of gliomas via serving as ceRNA and regulating the miR-194-5p/PC-esterase domain containing 1B (PCED1B) axis [28]. In this work, we confirmed that miR-215-3p was a direct target of PCED1B-AS1. According to previous reports, miR-215-3p is pivotal in cancer biology as a tumor suppressor [29,30]. This study revealed that miR-215-3p level was dramatically declined in GC tissues and cell lines, and miR-215-3p and PCED1B-AS1 expression were negatively correlated in GC tissues; the transfection of miR-215-3p mimics partially impeded the promoting effect of PCED1B-AS1 overexpression on the malignant biological behaviors of GC cells. These data suggest that PCED1B-AS1 can probably function as a ceRNA to regulate miR-215-3p and its downstream genes.

In the present work, CXCR1 was identified as a target gene of miR-215-3p in GC cells. CXCR1 mediates the progression of multiple cancers, including GC [15,18]. For example, IL-8 promotes the migration of liver cancer cells through CXCR1 and CXCR2, and targeting CXCR1/2 may be a strategy for treating liver cancer [31]. Another study reports that miR-761 can impede osteosarcoma cell growth and invasion partly via targeting CXCR1 [32]. In colorectal cancer cells, miR-215-3p improves the 5-Fu sensibility of cancer cells via regulating CXCR1 expression [16]. In GC, CXCR1 regulates the activation of AKT and ERK1/2 signal pathways to modulate the malignant biological behaviors of cancer cells [18]. Besides, E-cadherin is a cell adhesion molecule involved in cell-cell and cell-matrix interactions and EMT process of cancer cells, and it reduces the aggressiveness of cancer cells [33]; reportedly, CXCR1 knockdown increases E-cadherin expression in GC cells [18]. Herein, our rescue experiments revealed that inhibition of CXCR1 demonstrably restrained N-cadherin and Vimentin expressions, increased the expression of E-cadherin, and reversed the promoting effect of PCED1B-AS1 overexpression on the malignant biological behaviors of GC cells. In short, these data suggest that PCED1B-AS1 can facilitate the growth, migration, invasion and EMT of GC cells via modulating the miR-215-3p/CXCR1 axis.

## Conclusion

This study confirms the tumor-promoting effect of PCED1B-AS1 in GC. PCED1B-AS1 is highly expressed in GC cancer tissues and cell lines, and PCED1B-AS1 expression is associated with the clinicopathological characteristics of GC patients. Furthermore, PCED1B-AS1, as ceRNA, up-regulates CXCR1 through competitively binding to miR-215-3p to promote the malignancy of GC cells, suggesting that PCED1B-AS1/miR-215-3p/CXCR1 axis is a novel mechanism involved in GC progression.

## Data Availability

The data used to support the findings of this study are available from the corresponding author upon request.

## Highlights

1. LncRNA PCED1B-AS1 is up-regulated in gastric cancer tissues, and its high expression indicates poor prognosis.
2. PCED1B promotes the malignancy of GC cell lines.
3. PCED1B can increase the expression of CXCR1 via repressing miR-215-3p in GC cells.
